# ITRAQ-based quantitative proteomic analysis of *Fusarium moniliforme (Fusarium verticillioides)* in response to Phloridzin inducers

**DOI:** 10.1186/s12953-021-00170-2

**Published:** 2021-01-14

**Authors:** Rong Zhang, Weitao Jiang, Xin Liu, Yanan Duan, Li Xiang, Yanfang Wang, Yuanmao Jiang, Xiang Shen, Xuesen Chen, Chengmiao Yin, Zhiquan Mao

**Affiliations:** 1 State Key Laboratory of Crop Biology/College of Horticultural Science and Engineering, Shandong Agricultural University, Daizong Road No.61, Tai’an, 271018 Shandong China; 2 State Key Laboratory of Crop Biology, College of Agronomy, Shandong Agricultural University, Tai’an, 271018 Shandong China; 3 College of Chemistry and Material Science, Shandong Agricultural University, Tai’an, 271018 Shandong China

**Keywords:** *F. moniliforme*, Phloridzin, Mycelium proteomics, iTRAQ, Differential protein expression

## Abstract

**Background:**

Apple replant disease (ARD) has been reported from all major fruit-growing regions of the world, and is often caused by biotic factors (pathogen fungi) and abiotic factors (phenolic compounds). In order to clarify the proteomic differences of *Fusarium moniliforme* under the action of phloridzin, and to explore the potential mechanism of *F. moniliforme* as the pathogen of ARD, the role of *Fusarium* spp. in ARD was further clarified.

**Methods:**

In this paper, the quantitative proteomics method iTRAQ analysis technology was used to analyze the proteomic differences of *F. moniliforme* before and after phloridzin treatment. The differentially expressed protein was validated by qRT-PCR analysis.

**Results:**

A total of 4535 proteins were detected, and 293 proteins were found with more than 1.2 times (*P*< 0.05) differences. In-depth data analysis revealed that 59 proteins were found with more than 1.5 times (*P*< 0.05) differences, and most proteins were consistent with the result of qRT-PCR. Differentially expressed proteins were influenced a variety of cellular processes, particularly metabolic processes. Among these metabolic pathways, a total of 8 significantly enriched KEGG pathways were identified with at least 2 affiliated proteins with different abundance in conidia and mycelium. Functional pathway analysis indicated that up-regulated proteins were mainly distributed in amino sugar, nucleotide sugar metabolism, glycolysis/ gluconeogenesis and phagosome pathways.

**Conclusions:**

This study is the first to perform quantitative proteomic investigation by iTRAQ labeling and LC-MS/MS to identify differentially expressed proteins in *F. moniliforme* under phloridzin conditions. The results confirmed that *F. moniliforme* presented a unique protein profile that indicated the adaptive mechanisms of this species to phloridzin environments. The results deepened our understanding of the proteome in *F. moniliforme* in response to phloridzin inducers and provide a basis for further exploration for improving the efficiency of the fungi as biocontrol agents to control ARD.

**Supplementary Information:**

The online version contains supplementary material available at 10.1186/s12953-021-00170-2.

## Background

Apple is one of the most important fruit trees worldwide, and China has the largest area of apple tree cultivation. Apple replant disease (ARD) is widespread in China because repeated production in the same field is a common practice owing land scarcity and the replant of aged apple orchards. ARD causes the inhibition of root system development, stunts tree growth, and reduces yield and quality in replanted apple orchards [1–3], and is common in all of the major apple growing regions of the world [1]. The etiology of ARD is complex, many research results suggest that ARD is not attributed to only one factor, but rather to a combined effect of biotic factors and abiotic factors [4, 5]. Biotic factors including nematodes, bacteria, actinomycete, oomycetes and fungi species [6, 7], and most researches demonstrated fungal and oomycete genera were the main reason for ARD, i.e. fungal genera: *Fusarium* [5, 6, 8], *Rhizoctonia* [5, 6], *Cylindrocarpon* [3, 6, 9]; oomycete genera: *Phytophthora* [5, 6], *Pythium* [5, 6]. Abiotic factors such as soil structure, nutrition, and the release of allelochemicals through leaching, root exudation, volatilization, and/or decomposition of residues may also play roles in replant problems [10–12].

The harmful fungi were different in replant soil of different area’s orchards. Manici et al. [13] investigated three orchards in Germany, Austria and Italy, found that *Cylindrocarpon* spp. (*Ilyonectria* spp. and *Thelonectria* spp) is the main pathogenic fungus in three replanted orchards, while *pythium* spp. is just a pathogenic fungus in German. Van Schoor et al. [7] showed that pathogenic species of *Fusarium*, *Cylindrocarpon*, and *Pythium* were the primary obstacles to continuous cropping in replanted South African orchards. Kelderer et al. [14] showed that *Fusarium solani*, *F. oxysporum*, *binucleate Rhizoctoni*a spp. were the main soil-borne pathogens for ARD in Italy. So for, *F. moniliforme* also have been shown to be pathogenic on forest nurseries in Spain [15], Sorghum in India, Maize in Iowa State [16], Scots pine seedlings in Palencia [17], Peach in California [18] and apple in China [19]. *F. moniliforme* (*F. verticillioides*), *F. oxysporum*, and *F. proliferatum* isolated from replanted apple soil near the Bohai Bay region in China were highly pathogenic to seedlings of *Malus hupehensis* in our laboratory research cooperation studies [20, 21]. Phloridzin is a characteristic dihydrochalcone of the phenolic compounds produced by apple seedlings [11, 22–24], and phloridzin in the root exudates of *Malus x domestica* Borkh. and *Malus hupehensis* Rehd. might have consequences for research on the etiology of ARD [11, 12]. But how interaction of phloridzin (a phenolic compound) and *F. moniliforme* (a soil-borne pathogen) in the ARD is not yet fully understood.

In our study, we observed the effect of phloridzin on *F. moniliforme* on potato dextrose agar medium and inorganic salt culture medium, respectively. The effect of phloridzin on the proteome of *F. moniliforme* was further studied using iTRAQ technology. To the best of our knowledge, this is the first application of iTRAQ coupled nanoUHPLC-MS/MS technique to investigate the mechanism of *F. moniliforme* hyphae growth in response to phloridzin inducers. The primary aims of this research were to study: 1) Does phloridzin provide a carbon source for *F. moniliforme*? 2) How does the mycelium proteome of *F. moniliforme* change with phloridzin? 3) Which metabolic pathways of *F. moniliforme* have been changed by phloridzin?

## Methods

### Strains and culture conditions

The experimental processing settings were as follows: *F. moniliforme+*PDA(as CK), PDA medium + *F. moniliforme* + 1 ml phloridzin solution(as T). Stock cultures of the stains were prepared on potato dextrose agar (PDA) medium (20% potato extract, 2% glucose, 2% agar, pH 7.0). After being incubated for 7 days at 28 °C, mycelia were collected and stored at − 80 °C for protein preparation, respectively. Three biological replicates were used for both control and treatment in this study.

Our previous study found that phloridzin promoted the growth and division of *F. moniliforme* with the help of fluorescence labeling with SiC quantum dots (Fig. S1) [20]. The details of the experiment were as follows: The *F. moniliforme* was inoculated into Potato Dextrose (PD: 200 g potato and 20 g glucosein 1 L distilled water) liquid culture media and incubated in a thermostatic orbital shaker at 28 °C and 200 rpm for 48 h, and PD liquid culture media containing *F. moniliforme* was prepared. Three treatments were arranged: no phloridzin (T1: 45 mL PD liquid culture media containing *F. moniliforme* and 15 mL sterilized SiC quantum dots solution and 5 mL sterilized water). 0.5 mM phloridzin (T2: 45 mL PD liquid culture media containing *F. moniliforme* and 15 mL sterilized SiC quantum dots solution and 5 mL 0.5 mM phloridzin). 1.0 mM phloridzin (T3: 45 mL PD liquid culture media containing *F. moniliforme* and 15 mL sterilized SiC quantum dots solution and 5 mL 1.0 mM phloridzin), each treatment with 3 replicates. All samples of three treatments were incubated in a thermostatic shaker at 28 °C and 200 rpm for 3-40d. The growth and conidia division of *F. moniliforme* were observed at different time points under a fluorescence microscope. Living cell morphologies were observed and photographed using an Olympus IX-71 type fluorescence microscope (Japan).

In order to further verify whether phloridzin provides carbon source for *F. moniliforme*, inorganic salt culture medium (1.0 g of ammonium sulfate, 0.5 g of sodium chloride, 0.5 g of potassium dihydrogen phosphate, 1.5 g of dipotassium hydrogen phosphate, 0.2 g of magnesium sulfate, 20 g of agar, make up to 1000 ml with distilled water, and autoclave at 121 °C for 20 min) and phloridzin inorganic salt culture medium (1.0 g of ammonium sulfate, 0.5 g of sodium chloride, 0.5 g of potassium dihydrogen phosphate, 1.5 g of dipotassium hydrogen phosphate, 0.2 g of magnesium sulfate, 20 g of agar, 0.044 g of phloridzin, make up to 1000 ml with distilled water, and autoclave at 121 °C for 20 min) were set in this study.

### Protein preparation and sample preparation

Took a 2 g sample, added liquid extraction buffer and protease inhibitor in a certain ratio after grinding in liquid nitrogen in a frozen state, vortexed for 10 min. Added the same volume of Tris-saturated phenol (pH 8.0) and vortexed for 10 min; centrifuged at 12000 g at 4 °C for 20 min to obtain a phenol phase; took the phenol phase into a new centrifuge tube, added an equal volume of extraction buffer, vortexed Shake for 10 min; centrifuged at 12,000 g for 20 min at 4 °C to obtain a phenol phase; took the phenol phase into a new centrifuge tube, added the pre-chilled ammonium acetate methanol solution in proportion, and precipitated the protein overnight at − 20 °C; centrifuged at 12000 g for 20 min at 4 °C; discarded Supernatant, added 90% acetone and vortexed to mix and washed twice. Suspended the pellet with an appropriate volume of lysate (8 M urea + 1% SDS, protease inhibitor has been added) to fully dissolved the sample protein. Centrifuged at 4 °C, 12000 g for 20 min. Collected the supernatant. The quantitative results of BCA were shown in Fig. S2 and Table S1.

Lysis buffer (1% SDS, 8 M urea, 1x Protease Inhibitor Cocktail (Roche Ltd. Basel, Switzerland) was added into the samples. The lysis was performed by sonication on ice for 2 min and kept on ice for 30 min. After centrifugation at 15000 rpm for 15 min at 4 °C, the supernatant was collected and transferred to a new Eppendorf tube.

### Protein digestion and iTRAQ labeling

The protein concentration was determined by using the BCA protein assay, and then 100 μg of protein per condition was transferred into a new Eppendorf tube and the final volume was adjusted to 100 μL with 8 M urea. 2 μL of 0.5 M TCEP was added and the sample was incubated at 37 °C for 1 h, and then 4 μL of 1 M iodoacetamide was added to the sample and the incubation was last for 40 min protected from light at room temperature. After that, five volumes of − 20 °C pre-chilled acetone was added to precipitate the proteins overnight at − 20 °C. The precipitates were washed by pre-chilled 90% acetone aqueous solution for twice and then re-dissolved in 100 μL 100 mM TEAB. Sequence grade modified trypsin (Promega, Madison, WI) was added at the ratio of 1:50 (enzyme: protein, weight: weight) to digest the proteins at 37 °C overnight. The peptide mixture was desalted by C18 ZipTip, quantified by Pierce™ Quantitative Colorimetric Peptide Assay (23275) and then lyophilized by SpeedVac.

The resultant peptide mixture was labeled with iTRAQ 8Plex labeling kit (Sciex) following the manufacturer’s instructions. The labeled peptide samples were then pooled and lyophilized in a vacuum concentrator.

### RNA extraction and quantitative real-time polymerase chain reaction (qRT-PCR)

To validate differentially proteins, qRT-PCR was performed in triplicate using the same RNA samples as were used for the iTRAQ construction. An Fungal RNA Kit (Omega Bio-Tek, Norcross, Georgia, USA) was used to extract RNA. The concentration (ng/μL) and quality (A260/A280) of the total RNA were determined using a Nanodrop 2000 spectrophotometer (ThermoScientific, USA), and the integrity of the RNA was tested on an Agilent Technologies 2100 Bioanalyzer. First-strand cDNA was synthesized from 1 μg of total RNA using RevertAid First Strand cDNA Synthesis Kit (TransGEN, Beijing, China). SYBR® Green PCR Master Mix (TransGen) was then used for qRT-PCR. The β-actin was used as an internal control [25] and the relative quantification of specific mRNA levels was performed using the cycle threshold (Ct) 2^-ΔΔCt^ method (SoftwareIQ5 2.0) [26].

### High pH reverse phase separation

The peptide mixture was re-dissovled in the buffer A (buffer A: 20 mM ammonium formate in water, pH 10.0, adjusted with ammonium hydroxide), and then fractionated by high pH separation using Ultimate 3000 system (ThermoFisher scientific, MA, USA) connected to a reverse phase column (XBridge C18 column, 4.6 mm × 250 mm, 5 μm, (Waters Corporation, MA, USA). High pH separation was performed using a linear gradient, starting from 5% B to 45% B in 40 min (B: 20 mM ammonium formate in 80% ACN, pH 10.0, adjusted with ammonium hydroxide). The column was re-equilibrated at the initial condition for 15 min. The column flow rate was maintained at 1 mL/min and the column temperature was maintained at 30 °C. Twelve fractions were collected; each fraction was dried in a vacuum concentrator for the next step.

### nanoUHPLC-MS/MS analysis

The peptides were re-dissolved in solvent A containing 0.1% formic acid and analyzed by on-line nanospray LC-MS/MS on Q Exactive™ (Thermo Fisher Scientific, MA, USA) coupled to EASY-nano-LC 1200 system (Thermo Fisher Scientific, MA, USA). 2.5 μL peptide sample was loaded, and separated with 90 min-gradient, from 5 to 35% B (B: 0.1% formic acid in ACN). The column flow rate was maintained at 300 nL/min. The electrospray voltage of 2 kV versus the inlet of the mass spectrometer was used.

The mass spectrometer was run under data dependent acquisition mode, and automatically switched between MS and MS/MS mode. The parameters was: (1) MS: scan range (m/z)=300–1800; resolution=70,000; AGC target=3e6; maximum injection time=60 ms; include charge states=2–7; dynamic exclusion=20s; (2) HCD-MS/MS: resolution=17,500; isolation window=2.2; AGC target=5e4; maximum injection time=80 ms; collision energy=30.

### Proteomic data analysis

Tandem mass spectra were processed by PEAKS Studio version 8.5 (Bioinformatics Solutions Inc., Waterloo, Canada). PEAKS DB was set up to search the uniprot-proteome_UP000009096 databases (17,877 entries) assuming trypsin as the digestion enzyme. PEAKS DB were searched with a fragment ion mass tolerance of 0.05 Da and a parent ion tolerance of 10 ppm. Carbamidomethylation (C) and Itraq 8plex (K, N-term) were specified as the fixed modification. Oxidation (M), Deamidation (NQ) were specified as the variable modifications. Peptides were filter by 1% FDR and 1 unique. ANOVA was used for peptide and protein abundance calculation. Normalization was performed on averaging the abundance of all peptides. Medians were used for averaging. Different expressed proteins were filtered if their fold change were over 1.2 and contained at least 1 unique peptides with significance over 13 (*p*< 0.05).

### Bioinformatics analysis

The volcano plot, which plots significance versus fold-change on the y and x axes respectively, is a type of scatter-plot that is used to quickly identify changes in large data sets composed of replicate data. It is drawn by using ggplot2 package (http://ggplot2.org). Blast2GO version 5 was used for functional annotation, and GOATOOLS was used for functional enrichment of differential proteins. Blast2GO version 4 was used for functional annotation. Whole protein sequence database was analyzed by BlastP using whole database and mapped, annotated with gene ontology database. Statistically altered functions of different expressed proteins was calculated by Fisher’s exact test in BLAST2GO [27, 28]. KEGG Pathway analysis was processed by KOBAS (http://kobas.cbi.pku.edu.cn/) [29].

## Results

### Phloridzin provides carbon source for *F. moniliforme*

Our previous study found that phloridzin promoted the growth and division of *F. moniliforme*, The mycelial growth and conidia division of *F. moniliforme* were fast with the help of phloridzin, and division were faster in the 1.0 mM phloridzin than in the 0.5 mM phloridzin (Fig. S1) [20]. But how does phloridzin promote the growth of *F. moniliforme*? Provide carbon sources? In this study, the effects of phloridzin on *F. moniliforme* were further determined by plate culture and ITRAQ-based proteomics.

**Fig. 1:**
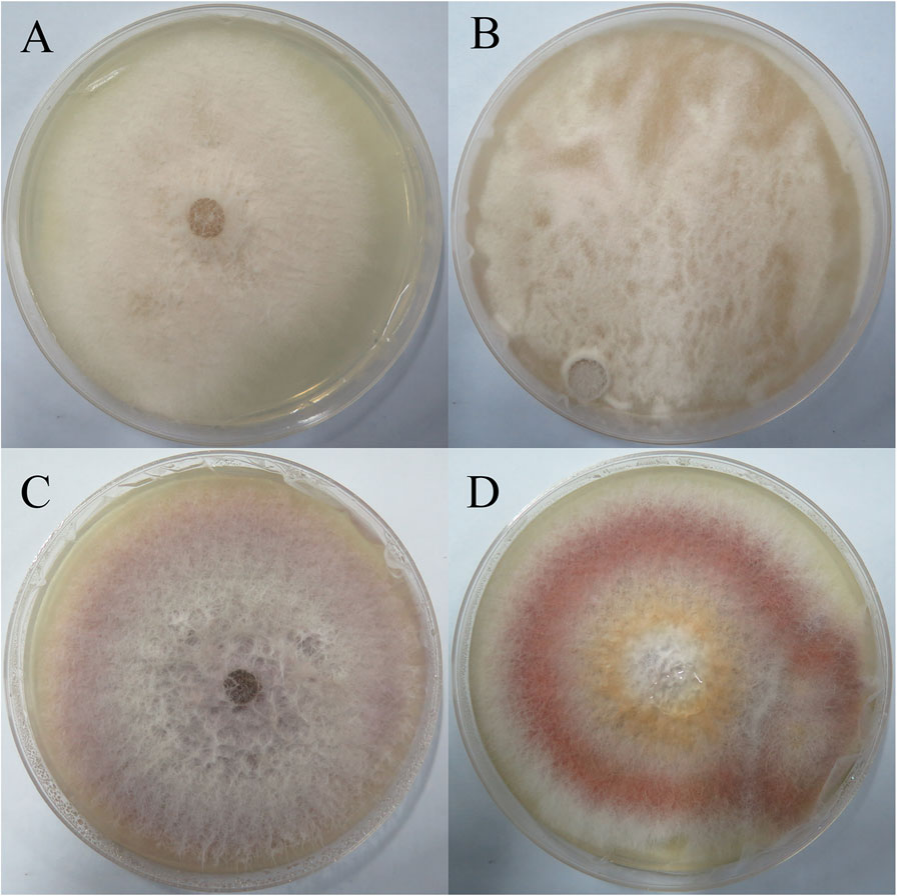
Growth of *F. moniliforme* on different media for 7 days. **a** inorganic salt culture medium; **b** phloridzin+inorganic salt culture medium; **c** Potato Dextrose Agar medium; **d** phloridzin+Potato Dextrose Agar medium

In order to further verify whether phloridzin provides carbon source for *F. moniliforme*, inorganic salt culture medium (without any carbon source) and phloridzin inorganic salt culture medium (phloridzin is the only carbon source) were set in this study. After 1–7 days of culture (Fig. 1a, b), it was found that *F. moniliforme* grew faster on the phloridzin inorganic salt medium than on the inorganic salt medium, which indicated that phloridzin provided carbon source for *F. moniliforme*. The color of *F. moniliforme* hyphae also changed under the action of phloridzin on PDA medium (Fig. 1c, d), which indicated that phloridzin had various effects on *F. moniliforme*. In order to further verify the effect of phloridzin on *F. moniliforme*, the cultured hyphae were sent to Jiachen Hengye (Beijing) Technology Limited company to determine the effect of phloridzin on the proteome of *F. moniliforme*.

### Overview of protein identification on ITRAQ technology

**Fig. 2:**
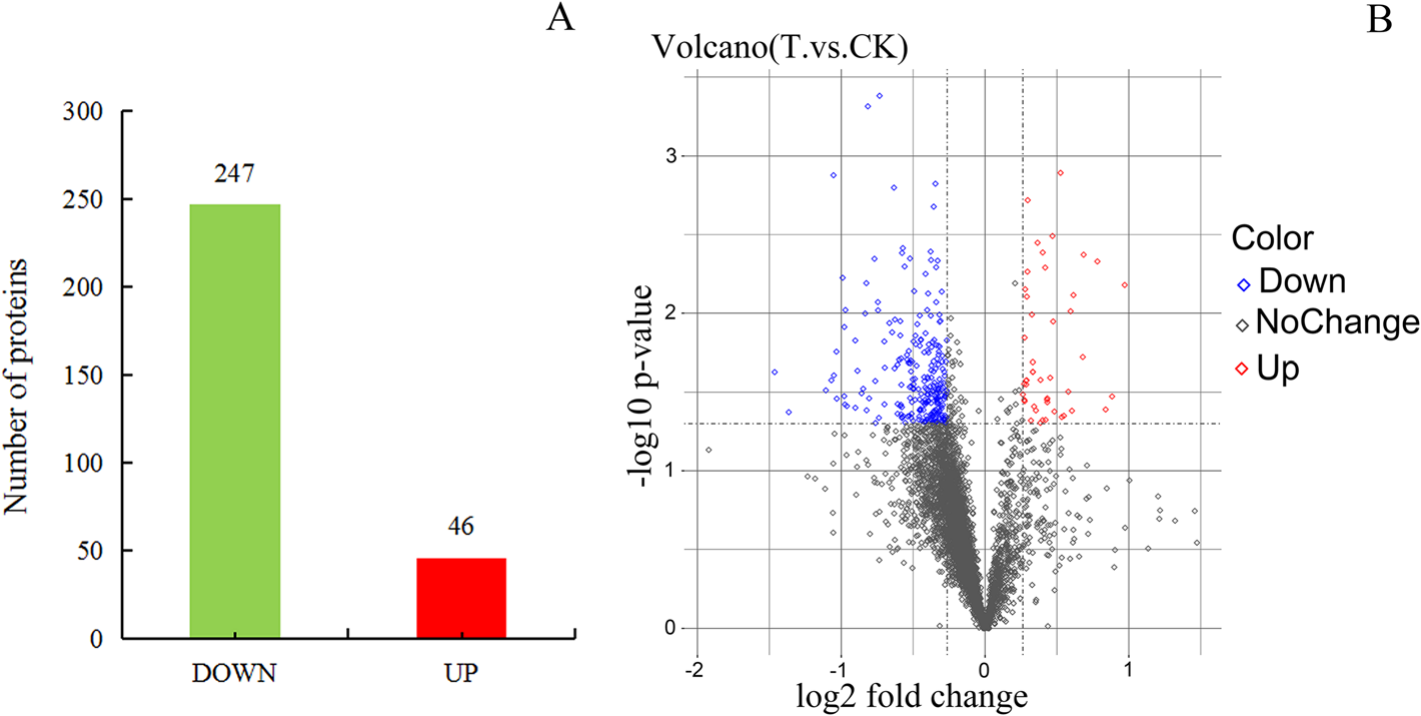
Analysis of differentially expressed proteins. **a** Numbers of up- and down-regulated proteins between the T.vs.CK inoculated at the collection. **b** Volcano Plot. Each point in the figure represents a protein, the abscissa represents the log2 (fold change) of the difference in the expression of the protein in the two groups, and the ordinate represents the negative logarithm of the *P* value of the change in the amount of protein expression. The blue dots represent the proteins down-regulate significantly, red dots represent the proteins up-regulate significantly, and gray dots are the proteins with no difference

The volcano map is a scatter plot with the log2 value of the Fold Change as the horizontal axis and the -log10 value of the *P* value as the vertical axis. According to the threshold of the significant change, the data is divided into three lines. The red dot is up, the blue dot is down, and the gray dot is unchanged. It can be seen in Fig. 2 that there are significant differentially expressed proteins in the T.vs.CK group, and there are more significant differentially expressed proteins with a more uniform distribution. In this project experiment, a total of 4535 Protein Groups were identified (Card value standard: Peptide Threshold 1.0% FDR, 1 Unique Peptide), and 293 proteins were found with more than 1.2 times (*P*< 0.05) differences.

Table 1 and Table 2 summarize the partial differential proteins by taking the difference fold value change more than 1.5 times as a significant upward adjustment, and using the difference fold value change less than − 1.5 as a significant downward adjustment change standard. It can be seen from Table 2 that there were 9 significantly up-regulated differential proteins, including Thiamine thiazole synthase(W7MF57), Protein NMT1(W7N1I1), and 7 proteins with unknown functional properties in the Uniport database. Among the above differential proteins, Thiamine thiazole synthase(W7MF57) protein closely related to *F. moniliforme* after phloridzin action.

**Table 1:** List of specific up-regulated proteins associated with *F. moniliforme* after phloridzin action

Uniprot No	Protein Name	Gene Name	Fold Change
W7MF57	Thiamine thiazole synthase	FVEG_09077	1.96
W7N1I1	Protein NMT1	FVEG_09760	1.84
W7LI78	Uncharacterized protein	FVEG_02082	1.79
W7MGT3	Uncharacterized protein	FVEG_10005	1.72
W7MVV3	Uncharacterized protein	FVEG_10707	1.61
W7LFH7	Uncharacterized protein	FVEG_00977	1.6
W7MW03	Uncharacterized protein	FVEG_12281	1.53
W7LPC0	Uncharacterized protein	FVEG_00987	1.52
W7MVN9	Uncharacterized protein	FVEG_13346	1.51

**Table 2:** List of specific down-regulated proteins associated with *F. moniliforme* after phloridzin action

UniProtKB.ID	Protein Name	Gene Name	Fold Change
W7LZI8_GIBM7	Uncharacterized protein	FVEG_03005	−1.5
W7N7H5_GIBM7	Aldo_ket_red domain-containing protein	FVEG_13653	−1.5
W7NGN0_GIBM7	Chitinase	FVEG_17546	−1.5
W7MX74_GIBM7	40S ribosomal protein S24	FVEG_11169	−1.5
W7M9V8_GIBM7	MFS domain-containing protein	FVEG_03769	−1.5
W7M6R7_GIBM7	DNA primase large subunit	FVEG_07421	−1.5
W7LY87_GIBM7	Eburicol 14-alpha-demethylase	FVEG_01123	−1.51
W7ME11_GIBM7	Uncharacterized protein	FVEG_08782	−1.51
W7N230_GIBM7	N-acetyltransferase domain-containing protein	FVEG_09917	−1.52
W7N9I2_GIBM7	EXPERA domain-containing protein	FVEG_11622	−1.52
W7M844_GIBM7	Uncharacterized protein	FVEG_03257	−1.52
W7MQY8_GIBM7	Inhibitor I9 domain-containing protein	FVEG_07283	−1.54
W7LN68_GIBM7	Glyco_hydro_cc domain-containing protein	FVEG_00761	−1.55
W7MTK8_GIBM7	Uncharacterized protein	FVEG_12915	−1.55
W7LZB4_GIBM7	D-fructose-6-phosphate amidotransferase	FVEG_01326	−1.56
W7LQM7_GIBM7	5-oxoprolinase (ATP-hydrolysing)	FVEG_03586	−1.59
W7MUP2_GIBM7	TPR_REGION domain-containing protein	FVEG_10371	−1.62
W7M0R4_GIBM7	Serine/threonine protein kinase	FVEG_03275	−1.62
A0A139YBS2_GIBM7	FAD-binding PCMH-type domain-containing protein	FVEG_13991	−1.63
W7N377_GIBM7	CPA1 family monovalent cation:H+ antiporter	FVEG_10281	−1.66
W7MZ75_GIBM7	Uncharacterized protein	FVEG_09142	−1.67
W7LP77_GIBM7	Uncharacterized protein	FVEG_00968	−1.67
W7MNY9_GIBM7	Chitinase	FVEG_08877	−1.68
W7N353_GIBM7	Uncharacterized protein	FVEG_10258	−1.7
W7NGJ7_GIBM7	Uncharacterized protein	FVEG_13470	−1.7
W7LYG9_GIBM7	Mannan polymerase II complex ANP1 subunit	FVEG_02813	−1.7
W7MKQ6_GIBM7	AB hydrolase-1 domain-containing protein	FVEG_10588	−1.75
W7MVA2_GIBM7	Uncharacterized protein	FVEG_10525	−1.76
W7MWT5_GIBM7	Uncharacterized protein	FVEG_10963	−1.77
W7MWW5_GIBM7	Uncharacterized protein	FVEG_13766	−1.77
W7LNY6_GIBM7	Uncharacterized protein	FVEG_00880	−1.78
W7MCX5_GIBM7	Uncharacterized protein	FVEG_06256	−1.8
W7LU13_GIBM7	Murein transglycosylase	FVEG_04460	−1.81
W7MRI3_GIBM7	MFS domain-containing protein	FVEG_12359	−1.85
W7M050_GIBM7	Uncharacterized protein	FVEG_03143	−1.87
W7MHE4_GIBM7	Endo-1,4-beta-xylanase	FVEG_07261	−1.87
W7N9Q1_GIBM7	Carboxylesterase 1	FVEG_11700	−1.95
W7MPD6_GIBM7	Uncharacterized protein	FVEG_11510	−1.96
W7MIF4_GIBM7	Uncharacterized protein	FVEG_07507	−1.96
FUM18_GIBM7	Sphingosine N-acyltransferase-like protein FUM18	FUM18	−1.97
W7MK62_GIBM7	Cytochrome b5 heme-binding domain-containing protein	FVEG_10665	−1.97
W7MBZ0_GIBM7	Uncharacterized protein	FVEG_08638	−1.99
W7MJV1_GIBM7	Uncharacterized protein	FVEG_10364	−2.04
W7N736_GIBM7	Uncharacterized protein	FVEG_13832	−2.05
W7MWN8_GIBM7	Uncharacterized protein	FVEG_13669	−2.07
W7MK12_GIBM7	AB hydrolase-1 domain-containing protein	FVEG_05956	−2.08
W7MT22_GIBM7	Serine/threonine protein kinase	FVEG_17286	−2.1
W7N7U0_GIBM7	Uncharacterized protein	FVEG_16989	−2.15
W7MUY6_GIBM7	Beta-xylanase	FVEG_13343	−2.58
W7MP35_GIBM7	Uncharacterized protein	FVEG_11455	−2.76

It can be seen from Table 2 that there were 50 significantly down-regulated differential proteins, including Beta-xylanase (W7MUY6), Serine/threonine protein kinase (W7MT22), Sphingosine N-acyltransferase-like protein FUM18 (FUM18), Carboxylesterase 1 (W7N9Q1), Endo-1 4-beta-xylanase (W7MHE4), Mannan polymerase II complex ANP1 subunit (W7LYG9), Chitinase (W7MNY9, W7NGN0), CPA1 family monovalent cation:H+antiporter (W7N377), Serine/threonine protein kinase (W7M0R4), Murein transglycosylase (W7LU13), 5-oxoprolinase (ATP-hydrolysing, W7LQM7), D-fructose-6-phosphate amidotransferase (W7LZB4), Eburicol 14-alpha-demethylase (W7LY87), DNA primase large subunit (W7M6R7), 40S ribosomal protein S24 (W7MX74), 12 proteins with domain-containing protein and 22 proteins with unknown functional properties in the Uniport database. Among the above differential proteins, Beta-xylanase (W7MUY6), Serine/threonine protein kinase (W7MT22), Sphingosine N-acyltransferase-like protein FUM18 (FUM18), Carboxylesterase 1 (W7N9Q1), Endo-1 4-beta-xylanase (W7MHE4), annan polymerase II complex ANP1 subunit (W7LYG9), Chitinase (W7MNY9, W7NGN0), Murein transglycosylase (W7LU13), 5-oxoprolinase (ATP-hydrolysing, W7LQM7), D-fructose-6-phosphate amidotransferase (W7LZB4), Eburicol 14-alpha-demethylase (W7LY87) protein closely related to *F. moniliforme* after phloridzin action.

**Fig. 3:**
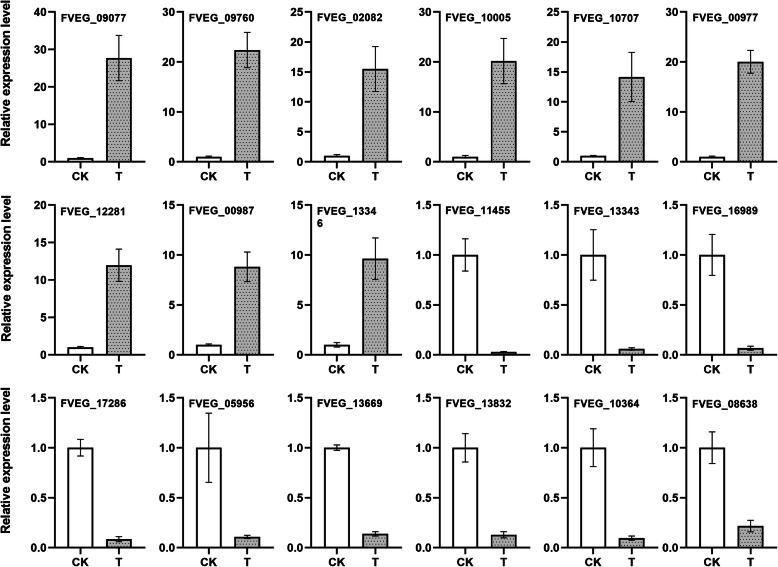
Comparison of qRT-PCR analysis for eighteen selected proteins with different abundance identified by iTRAQ data. Changes in the transcript (qRT-PCR analysis) and protein abundance (iTRAQ analysis) of T and CK were compared. For iTRAQ data analysis and qRT-PCR data analysis, details can be found in the Materials and methods section. The qRT-PCR data represent the mean ± S.D. (standard deviation) of three biological replicates. All primers and gene abbreviations are listed in Table S2

Based on methods of quantitative proteomics iTRAQ analysis, qRT-PCR identified 9 up-regulated proteins and 9 down-regulated proteins. Among the 9 up-regulated proteins, most proteins were basically consistent with the result of qRT-PCR. Although there were some quantitative differences in expression level, the trend of expression level was similar in the data between iTRAQ and qRT-PCR. We found that most of the 9 down-regulated proteins were highly consistent with the expression levels obtained by qRT-PCR. Primers for qRT-PCR and protein reference number are listed (Table S2, Fig. 3).

### GO enrichment analysis of differential proteins

**Fig. 4:**
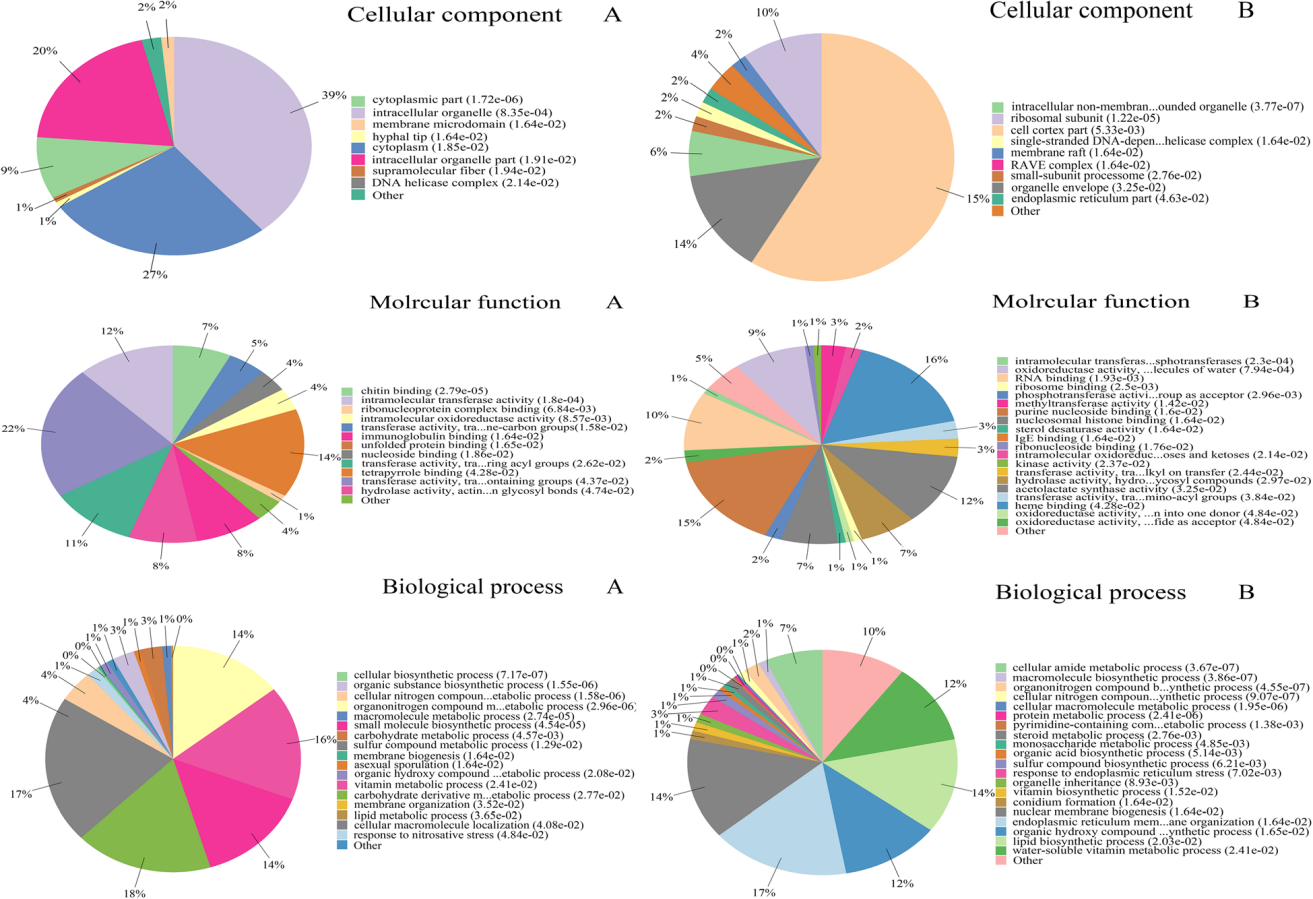
Gene ontology enrichment analysis of differentially expressed proteins. **a** The proportion of protein number enriched in the first 20 items with the lowest *P*-value among the three classification enrichment results of Biological process, Molecular function and Cellular component at Level 3; **b** The proportion of protein number enriched in the first 20 items with the lowest P-value among the three classification enrichment results of Biological process, Molecular function and Cellular component at Level 4

GO functional annotation is mainly divided into three categories: Biological Process (BP), Molecular Function (MF) and Cellular Component (CC). The summarized GO mapping and annotation data of differential proteins at GO level3 and level4 were shown in Fig. 4a, b, respectively. Figure 4 showed that differential proteins were mainly enrich in cell cortex part (GO:0044448), intrracellular organelle (GO:0043229), cytoplasm (GO:0005737), intrracellular organelle part (GO:0044446), organelle envelope (GO:0031967), ribosomal subunit (GO:0044391) of cellular component. In biological process, differential proteins were mainly distributed in carbohydrate derivative metabolic process (GO:1901135), sulfur compound metabolic process(GO:0006790), endoplasmic reticulum membrane organization(GO:0090158), vitamin biosynthetic process(GO:0009110), small molecule biosynthetic process(GO:0044283), organonitrogen compound metabolic process(GO:1901564), nuclear membrane biogenesis(GO:0101025), lipid biosynthetic process(GO:0008610), organic hydroxy compound biosynthetic process(GO:1901617) and water-soluble vitamin biosynthetic process(GO:0042364). While under the category of molecular function, differential proteins were mostly related to transferase activity, transferring phosphorus-containing groups(GO:0016772), heme binding(GO:0020037), purine nucleoside binding(GO:0001883), tetrapyrrole binding(GO:0046906), intramolecular transferase activity(GO:0016866), nucleosomal histone binding(GO:0031493), transferase activity, transferring acyl groups(GO:0016746) and RNA binding(GO:0003723).

### KEGG pathway enrichment analysis of differential proteins

To identify the biological pathway information of *F. moniliforme* in response to Phloridzin inducers, these 293 proteins were further mapped to the corresponding pathways included in the KEGG database. These 293 proteins could be mapped to 64 pathways. Among these metabolic pathways, a total of 8 significantly enriched KEGG pathways were identified with at least 2 affiliated proteins with different abundance in conidia and mycelium (Table 3) when the *p*-value was set at 0.05. Ribosome and Biosynthesis of antibiotics were the first and second significantly enriched pathways, respectively. Other representative pathways included Amino sugar and nucleotide sugar metabolism, Glycolysis / Gluconeogenesis and Phagosome were also significantly enriched pathways.

**Table 3:** KEGG pathway enrichment analysis of proteins with different abundance

KEGG Pathway	Pathway ID	Number of proteins	*P*-Value
Ribosome	fpu03010	32	1.05904E-17
Amino sugar and nucleotide sugar metabolism	fpu00520	8	0.015900092
Glycolysis / Gluconeogenesis	fpu00010	7	0.023264668
Biosynthesis of antibiotics	fpu01130	21	0.032523714
Phagosome	fpu04145	5	0.039364782
Steroid biosynthesis	fpu00100	4	0.039975385
Pantothenate and CoA biosynthesis	fpu00770	3	0.042509802
Thiamine metabolism	fpu00730	2	0.043935149

**Fig. 5:**
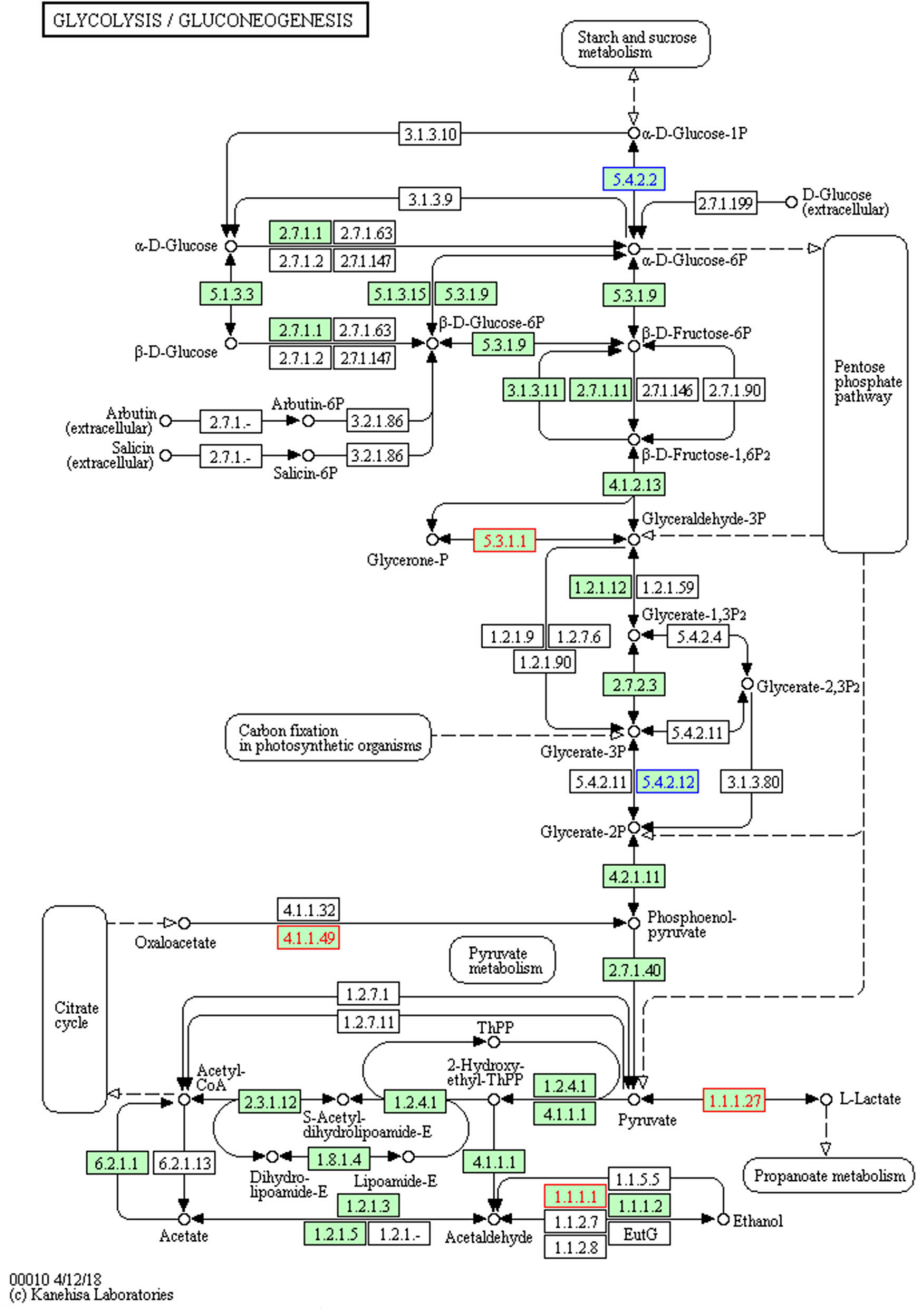
KEGG pathway enriched by differentially expressed proteins. Blue box nodes indicate down regulation and red box nodes indicate up regulation

As shown in Fig. 5, the expression levels of glycolysis / gluconeogenesis-related proteins in *F. moniliforme* were 2 down-regulated and 4 up-regulated. Moreover, it was found that the differentially expressed proteins W7N5X1, W7MA30, W7M3M6, and W7MPI9, which collectively show significantly up-regulation, participate in 19 metabolic pathways in total, and the differentially expressed differentially-formed proteins W7LNK6, W7M651, participate in 13 metabolic pathways.

**Fig. 6:**
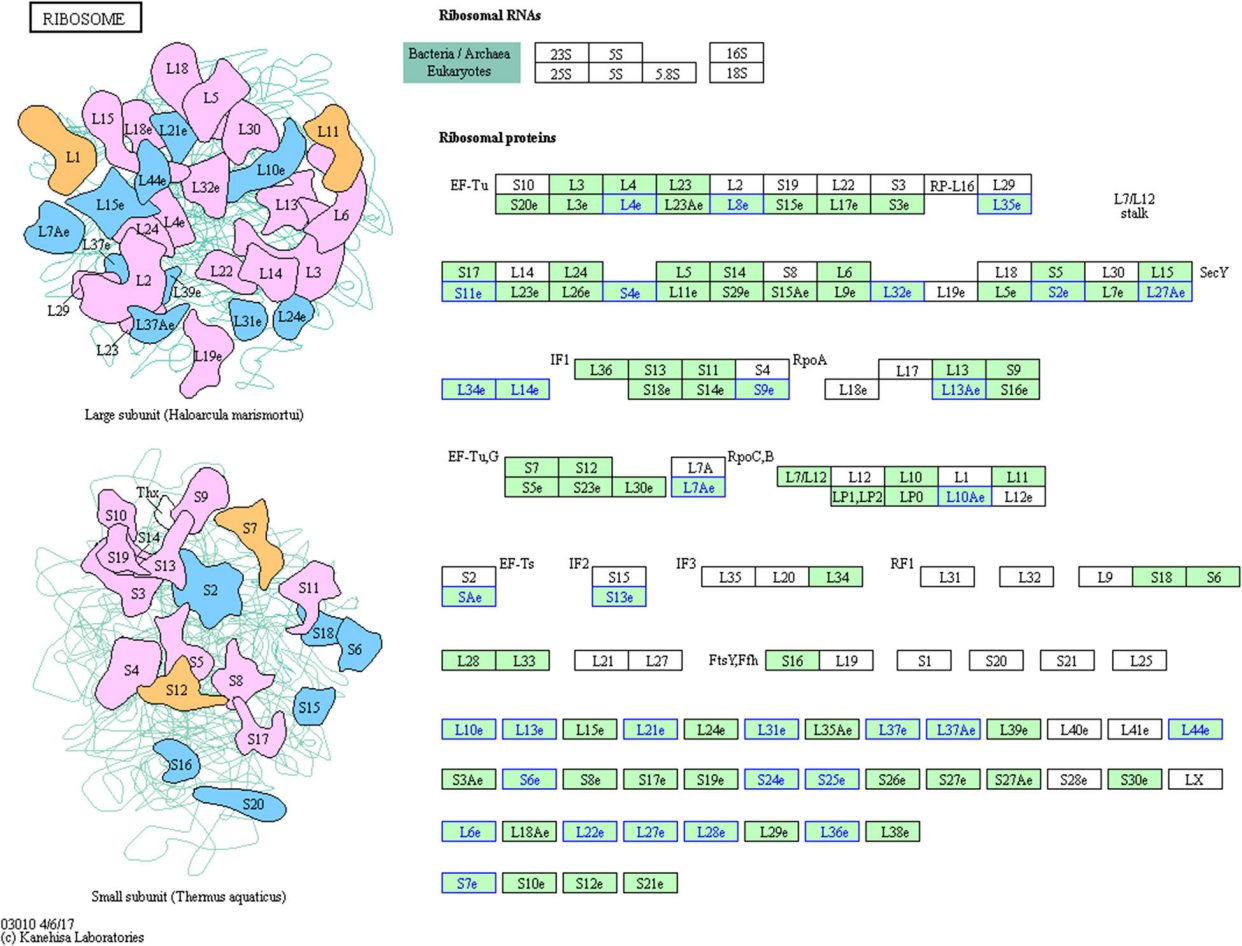
KEGG ribosome pathway enriched by differentially expressed proteins. Blue box nodes indicate differentially expressed proteins

Ribosome proteins can affect and regulate biological processes such as gene transcription, translation, cell proliferation and apoptosis. In this omics experiment, we used the Kanehisa database to analyze the differential proteins detected by the ITRAQ quantitative proteomics method. A total of 32 ribosome-associated proteins were found to have significant differential expression. These ribosomal proteins are structurally distributed in large ribosomal subunits and small ribosomal subunits (Fig. 6). Among them, the expression levels of ribosomal-related proteins in *F. moniliforme* are all down-regulated, showing a huge change in expression levels.

## Discussion

Primary fungal and oomycete genera reported as containing species that were pathogenic toward apple trees, include the fungal genera *Cylindrocarpon*, *Fusarium* and *Rhizoctonia*, and the oomycete genera *Phytophthora* and *Pythium* [3, 30]. *Fusarium* sp. was a significant plant pathogen, which had the diversity of affected hosts, numbers of pathogen taxa, and types of habitats in plant pathology [31]. *Fusarium* isolates, such as *F. tricinctum* (Corda) Sacc, *F. graminearum*, *F. solani* (Mart.) Sacc. and *F. avenaceum* (Fr.) Sacc. have been shown to be pathogenic [3, 6, 32–39]. And *F. moniliforme* have also been shown to be pathogenic on forest nurseries in Spain [15], Sorghum in India, Maize in Iowa State [16], Scots pine seedlings in Palencia [17], Peach in California [18] and apple in China [19, 21]. After *Fusarium* contacts the host plant, it initiates physiological and biochemical reactions inside the strain through processes such as surface molecular interaction and signal transmission, and produces metabolites that act on the plant body to cause pathogenic effects. It mainly includes cell wall degrading enzymes, toxins, growth regulators and their analogues.

Thiamine has a relatively important role in the body, and it has an effect on the growth of bacteria and amino acid metabolism. Thiamine is an important enzyme co-factor in all organisms. It has four forms in the body: thiamine triphosphate (TTP), bisamine (pyrophosphate) thiamine (TPP / TDP), single Thiamine Phosphate (TMP), Free Thiamine (FT). TPP plays a key role in the metabolism of amino acids and carbohydrates in the body (glycolytic pathway, tricarboxylic acid cycle, pentose phosphate cycle, etc.). Thiazole synthase is a rate-limiting enzyme for thiamine synthesis, and is involved in the synthesis of thiamine, and its function is related to the growth of bacteria and spore formation. In this test, a large amount of thiamine biosynthetic enzyme expression in *F. moniliforme* under phloridzin environment is beneficial to the synthesis of thiamine in *F. moniliforme*, and then promotes the growth of *F. moniliforme* and spore formation. It is consistent with the previous research results of this research group [20]. Phloridzin was utilized directly as a sugar alternative and a toxin accelerator by *Valsa mali* [40]. Legumes can co-exist with rhizobium to form a nodule, and the cell group containing the rhizobium in the nodule forms a bacterial-containing tissue. Recent studies have reported that exogenous thiamine can increase the diameter of root nodules, which indicates that leguminous plants and rhizobium treated with exogenous thiamine have more active division and differentiation of nodule-forming cells [41].

To identify the biological pathway information of *F. moniliforme* in response to Phloridzin inducers, these 293 proteins were further mapped to the corresponding pathways included in the KEGG database. These 293 proteins could be mapped to 64 pathways. Among these metabolic pathways, a total of 8 significantly enriched KEGG pathways were identified with at least 2 affiliated proteins with different abundance in conidia and mycelium when the *p*-value was set at 0.05. Ribosome and Biosynthesis of antibiotics were the first and second significantly enriched pathways, respectively. Other representative pathways included Amino sugar and nucleotide sugar metabolism, Glycolysis / Gluconeogenesis and Phagosome were also significantly enriched pathways. Phosphoenolpyruvate carboxykinase is widely present in animals, plants, microorganisms, and cells. It catalyzes the conversion of oxaloacetate to phosphoenolpyruvate and is a key enzyme that regulates the gluconeogenesis pathway. Gluconate 5-dehydrogenase is a coenzyme-dependent reductase that belongs to the short-chain dehydrogenase SDR family. It can catalyze D-gluconic acid and 5-keto-D-gluconic acid (5 -KGA) reversible redox conversion, so as to regulate the conversion of carbon source and reducing power in the body to maintain metabolic balance in the body, has an important role in glucose metabolism [42, 43]. According to GO annotation classification, most proteins are related to carbon metabolism. In biological process, differential proteins were mainly distributed in carbohydrate derivative metabolic process (GO:1901135), sulfur compound metabolic process(GO:0006790), vitamin biosynthetic process(GO:0009110), organonitrogen compound metabolic process(GO:1901564). While under the category of molecular function, differential proteins were mostly related to transferase activity, transferring phosphorus-containing groups(GO:0016772), heme binding(GO:0020037), transferase activity, transferring acyl groups(GO:0016746). In this study, the large expression of phosphoenolpyruvate carboxykinase and 5-gluconate dehydrogenase in *F. moniliforme* under the phloridzin environment is helpful to promote the gluconeogenesis pathway in *F. moniliforme* Amino acid metabolism and carbohydrate metabolism. In addition, 3-methyl-2-oxobutanoate hydroxymethyltransferase, phosphopantothenoylcysteine decarboxylase, acyl-CoA dehydrogenase, triosephosphate isomerase, related to beta-1,4-mannosyltransferase, related to gamma-glutamyltransferase, alcohol dehydrogenase 1, L-lactate dehydrogenase also up-regulated, L-lactate dehydrogenase is a key enzyme that produces lactic and phenyllactate [44].

β-1,4-xylanase is a key enzyme in the process of xylan degradation. Caleronieto et al. [45] inactivated xylR gene and xylR gene in *Fusarium oxysporum* tomato specialization, and found that the knockout of xlnR can make the expression of XYL3 and XYL genes Decreased, xylanase activity was correspondingly reduced, but the virulence was not weakened. Analysis may be because the knockout of xlnR did not completely eliminate the xylanase activity, and the remaining enzyme activity was still sufficient to produce a △ xlnR mutant strain. Virulence. Garcia-Maceira et al. [46] used an inactive allele to replace the target gene and found that the pathogenicity of the XYL3 mutant variant of the xylanase gene is similar to that of the wild-type strain, indicating that the XYL3 gene has Pathogenicity does not play a key role. Therefore, xylanase and cellulase may not play a key role in the pathogenicity of pathogenic bacteria. In this test, the expression of endo-1,4-beta-xylanase C and endo-1,4-beta-xylanase in *F. moniliforme* under phloridzin environment was down-regulated, which may be caused by xylanase degradation the accumulation of xylose in the culture medium, the accumulation of xylose inhibited the secretion of xylanase. Many regulatory genes in *F. moniliforme* infection may exist in multiple metabolic pathways at the same time, function in multiple biological processes, and constitute a complex biological system involving pathogens and hosts, proteins within pathogens, and proteins and Other complex inter-molecular networks of interactions [47]. Therefore, protein-based interaction networks based on pathogens will help to further understand the mechanism of pathogen infection.

## Conclusions

This study is the first to perform quantitative proteomic investigation by iTRAQ labeling and LC-MS/MS to identify differentially expressed proteins in *F. moniliforme* under phloridzin conditions. The results confirmed that *F. moniliforme* presented a unique protein profile that indicated the adaptive mechanisms of this species to phloridzin environments. Differentially expressed proteins were influenced a variety of cellular processes, particularly metabolic processes. Among these metabolic pathways, a total of 8 significantly enriched KEGG pathways were identified with at least 2 affiliated proteins with different abundance in conidia and mycelium. Functional pathway analysis indicated that up-regulated proteins were mainly distributed in amino sugar, nucleotide sugar metabolism, glycolysis/ gluconeogenesis and phagosome pathways. The results deepened our understanding of the proteome in *F. moniliforme* in response to phloridzin inducers and provide a basis for further exploration for improving the efficiency of the fungi as biocontrol agents to control ARD.

## Supplementary Information

**Additional file 1:** **Fig. S1.** F. moniliforme labeled with the SiC quantum dots in different concentrations of the phloridzin and observed with fluorescence microscopy (200 ×) on the 40 th day. (a) No phloridzin, T1. (b) 0.5 mM phloridzin, T2. (c) 1.0 mM phloridzin, T3. **Fig. S2.** Protein standard curve. Dilute protein standards with ultrapure water into samples of different concentrations (0.0 mg·mL^-1^, 0.1 mg·mL^-1^, 0.2 mg·mL^-1^, 0.3 mg·mL^-1^, 0.4 mg·mL^-1^, 0.5 mg·mL^-1^, 0.6 mg·mL^-1^). According to the BCA kit instructions, draw the standard curve with protein content as the abscissa and absorbance as the ordinate. The standard equation was y=1.1872x-0.1164, and the correlation coefficient R^2^=0.9989. **Table S1.** Sample protein quantification results. **Table S2.** Primer sequencesused for the qRT-PCR validation of selected differentially expressed proteins and β-actin.

## Data Availability

The datasets supporting the conclusions of this article are included within the article and its additional files.
