# Supplementary material for: ITRAQ-based quantitative proteomic analysis of Fusarium moniliforme (Fusarium verticillioides) in response to Phloridzin inducers
Source: Proteome Sci. 2021 Jan 14;19:2. doi: 10.1186/s12953-021-00170-2 (PMC7807804; doi:10.1186/s12953-021-00170-2)
Supplement: Supplementary file 1 — Additional file 1: Fig. S1. F. moniliforme labeled with the SiC quantum dots in different concentrations of the phloridzin and observed with fluorescence microscopy (200 ×) on the 40 th day. (a) No phloridzin, T1. (b) 0.5 mM phloridzin, T2. (c) 1.0 mM phloridzin, T3. Fig. S2. Protein standard curve. Dilute protein standards with ultrapure water into samples of different concentrations (0.0 mg·mL-1, 0.1 mg·mL-1, 0.2 mg·mL-1, 0.3 mg·mL-1, 0.4 mg·mL-1, 0.5 mg·mL-1, 0.6 mg·mL-1). According to the BCA kit instructions, draw the standard curve with protein content as the abscissa and absorbance as the ordinate. The standard equation was y=1.1872x-0.1164, and the correlation coefficient R2=0.9989. Table S1. Sample protein quantification results. Table S2. Primer sequencesused for the qRT-PCR validation of selected differentially expressed proteins and β-actin. [file 12953_2021_170_MOESM1_ESM.doc]

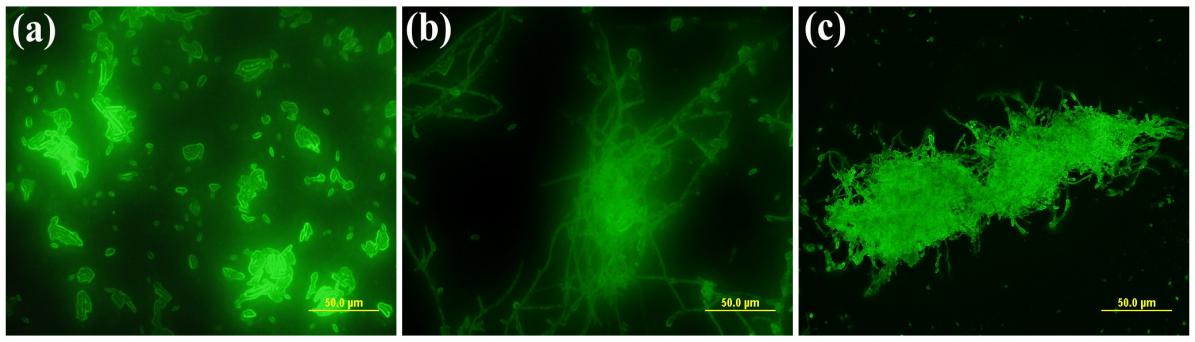


**Fig. S1** *F. moniliforme* labeled with the SiC quantum dots in different concentrations of the phloridzin and observed with fluorescence microscopy (200 ×) on the 40 th day. (a) No phloridzin, T1. (b) 0.5 mM phloridzin, T2. (c) 1.0 mM phloridzin, T3.


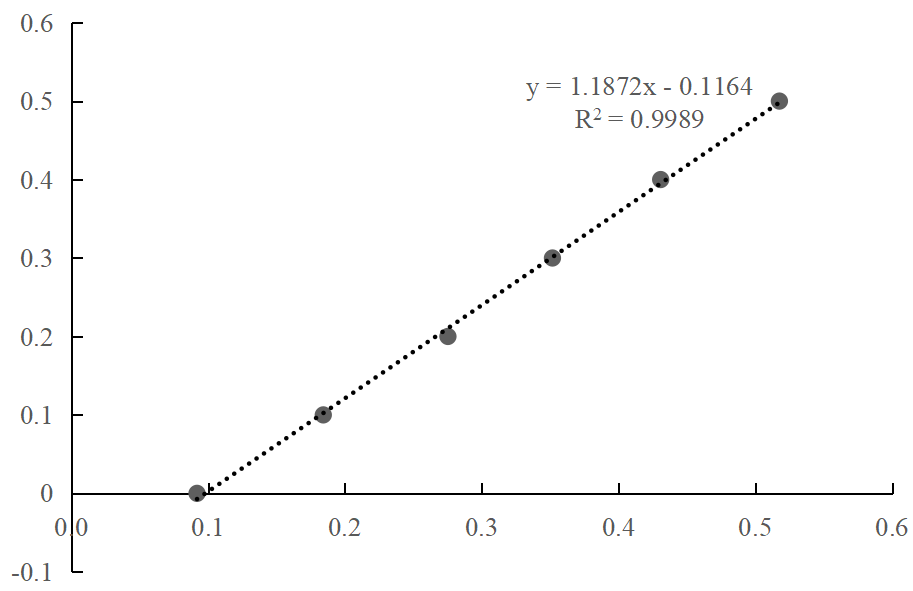


**Fig.S2** Protein standard curve. Dilute protein standards with ultrapure water into samples of different concentrations (0.0 mg·mL^-1^, 0.1 mg·mL^-1^, 0.2 mg·mL^-1^, 0.3 mg·mL^-1^, 0.4 mg·mL^-1^, 0.5 mg·mL^-1^, 0.6 mg·mL^-1^). According to the BCA kit instructions, draw the standard curve with protein content as the abscissa and absorbance as the ordinate. The standard equation was y=1.1872x-0.1164, and the correlation coefficient R^2^=0.9989.

**Table S1 Sample protein quantification results.**

| Number | Sample name | Protein concentration（mg·mL^-1^） | Total protein(mg) |
| --- | --- | --- | --- |
| 1 | CK1 | 12.46 | 3.12 |
| 2 | CK2 | 8.45 | 2.11 |
| 3 | CK3 | 10.09 | 2.52 |
| 4 | T-1 | 8.02 | 2.01 |
| 5 | T-2 | 6.37 | 1.59 |
| 6 | T-3 | 9.97 | 2.49 |

**Table S2 Primer sequencesused for the qRT-PCR validation of selected differentially expressed proteins and β-actin.**

|  | Uniprot No | Protein Name | Gene Name | Gene ID | Fold Change | Forward primer (5′-3′) | Reverse primer (5′-3′) | RT-PCR Ratio (T/CK) |
| --- | --- | --- | --- | --- | --- | --- | --- | --- |
| UP-regulated | W7MF57 | Thiamine thiazole synthase | FVEG_09077 | XP_018755777.1 | 1.96 | GGTCGGTGTTCCTTATGA | ATGATGGTAGAGGTGAAGAG | 27.69 |
|  | W7N1I1 | Protein NMT1 | FVEG_09760 | XP_018756779.1 | 1.84 | ATCGGCTATGTCGGTGAA | TGTAGTCGTCAGGAGTCAT | 22.38 |
|  | W7LI78 | Uncharacterized protein | FVEG_02082 | XP_018745278.1 | 1.79 | GAACCGATCAGTACAAGAGT | GGCATTCCAGAGTTATTCAC | 15.48 |
|  | W7MGT3 | Uncharacterized protein | FVEG_10005 | XP_018757074.1 | 1.72 | GCTAAACCAGGCAATCTATG | GATGTCGCTTACCGCTAA | 20.17 |
|  | W7MVV3 | Uncharacterized protein | FVEG_10707 | XP_018758026.1 | 1.61 | GACTACCGACTGCTACAT | GTCCTTGATACCGTAATACC | 14.16 |
|  | W7LFH7 | Uncharacterized protein | FVEG_00977 | XP_018743515.1 | 1.6 | GGATGGGATAGAGTAAGTTG | GATACATTCAGACGCTACC | 20.03 |
|  | W7MW03 | Uncharacterized protein | FVEG_12281 | XP_018761642.1 | 1.53 | GAATCATCGCTCAAGGCT | ATCCATCTCACGCTTCATAC | 11.96 |
|  | W7LPC0 | Uncharacterized protein | FVEG_00987 | XP_018743531.1 | 1.52 | TAACACGACGCTGGATAG | AGGCTGTCAGGATAGAGA | 8.81 |
|  | W7MVN9 | Uncharacterized protein | FVEG_13346 | XP_018761522.1 | 1.51 | GCTAAGGCTAATCAGGAG | CTACTTCTCAAGGCAGAC | 9.62 |
| DOWN-regulated | W7MP35 | Uncharacterized protein | FVEG_11455 | XP_018759037.1 | -2.76 | CACGGTTGCTATCCTATCA | CTTGGCTGAAGAGACTGA | 0.03 |
|  | W7MUY6 | Beta-xylanase | FVEG_13343 | XP_018761519.1 | -2.58 | CATTGGTATCACCGTCTG | GTAGTTGGAGTCGAACAG | 0.06 |
|  | W7N7U0 | Uncharacterized protein | FVEG_16989 | XP_018758723.1 | -2.15 | CTTGTTGACTACCACGAC | ACAGCTACCGCAAGTCTT | 0.07 |
|  | W7MT22 | Serine/threonine protein kinase | FVEG_17286 | XP_018760447.1 | -2.1 | CCTGTCAATTCTCAACCATCTC | TGTGTAAGTCTCCGCTTGT | 0.08 |
|  | W7MK12 | AB hydrolase-1 domain-containing protein | FVEG_05956 | XP_018751203.1 | -2.08 | GACAAGCAAACTCCTACTGA | CCTCTTCGGCATAATAGCA | 0.11 |
|  | W7MWN8 | Uncharacterized protein | FVEG_13669 | XP_018761909.1 | -2.07 | GGACAATCCAGTCTCTCT | CGGTGTTGATCTTGAAGTTC | 0.14 |
|  | W7N736 | Uncharacterized protein | FVEG_13832 | XP_018762091.1 | -2.05 | CAGCATCATCGGTCTCAA | CAGTGGCGTGACATTATCT | 0.13 |
|  | W7MJV1 | Uncharacterized protein | FVEG_10364 | XP_018757562.1 | -2.04 | ACTACACCGTCACCTTCAA | CATCAGGTCCGATCTTGTTC | 0.10 |
|  | W7MBZ0 | Uncharacterized protein | FVEG_08638 | XP_018755204.1 | -1.99 | GTGCTGGTCTGCTCATAA | TCGTCACTTGGACAGTCA | 0.22 |
| β-actin |  |  |  |  |  | ATGGTCAAGGCCGGTTTCG | TCAGGATGCCTCTCTTGGCC |  |
